# Use of food-grade enzymes to obtain milk proteins hydrolysates with hypoallergenic properties

**DOI:** 10.1186/2045-7022-3-S3-P8

**Published:** 2013-07-25

**Authors:** I Herranz, E Molina, I López Expósito

**Affiliations:** 1 Bioactivity and Food Analysis, Institute for Food Science Research (CIAL) (CSIC-UAM), Madrid, Spain

## Background

Food is the most frequent cause of allergy during the early childhood, and possibly the only trigger within the first two years of life. The foods commonly involved in are cow’s milk and hen’s egg. So far, strict avoidance of the responsible food in any of its forms is usually the only available treatment. Thus, obtaining new hypoallergenic foods or food ingredients derived from milk or egg to be consumed directly or added to other foods would be of great benefit for the different stakeholders (patients, family, industries, caterings, etc). A strategy to reduce food allergenicity relies within its own processing. It is well known that food processing induces important structural changes that may affect protein allergenicity. Those changes might be due to epitope destruction, easier access to epitopes due to protein unfolding, etc. Technological treatments such as hydrolysis have been extensively studied, but its potential use to produce hypoallergenic food need more research.

The purpose of this study was to test different enzymes commonly used in the food industry to obtain hypoallergenic hydrolysates from milk proteins.

## Methods

Principal allergens from milk, β-casein (β-CN) and β-lactoglobulin (β-Lg), were hydrolysed with Alcalase, Neutrase and Bromelain at different time points. Hydrolysates were analyzed by SDS-PAGE and RP-HPLC. Selected samples were tested against 6 patients’ IgE sera by inhibition ELISA respectively.

## Results

Higher values of IC50 for β-CN and β-Lg hydrolysates were obtained at higher incubation times when Alcalase or Neutrase were employed as enzymes. β-CN is hydrolyzed more efficiently showing higher IC50 values than β-Lg, at similar incubation times. Between them, Neutrase produced hydrolysates with lower allergenicity.

On the other hand, Bromelain did not have any influence on β-Lg hydrolysis or allergenicity, but it showed a discrete effect on β-CN hydrolysis with a moderate influence on its allergenicity.

## Conclusion

Neutrase is a promising enzyme in order to obtain hypoallergenic hydrolysates from β-casein.

## Disclosure of interest

None declared.

